# The invasion depth measurement of bladder cancer using T2-weighted magnetic resonance imaging

**DOI:** 10.1186/s12938-020-00834-8

**Published:** 2020-12-07

**Authors:** Yang Liu, Haojie Zheng, Xiaopan Xu, Xi Zhang, Peng Du, Jimin Liang, Hongbing Lu

**Affiliations:** 1 School of Biomedical Engineering, Air Force Medical University, No. 169 Changle West Road, Xi’an, Shaanxi 710032 PR China; 2 School of Life Sciences and Technology, Xidian University, 266 Xinglong Section of Xifeng Road, Xi’an, Shaanxi 710126 PR China

**Keywords:** Bladder cancer, Support vector machine, Feature selection, Segmentation, Invasion depth

## Abstract

**Background:**

Invasion depth is an important index for staging and clinical treatment strategy of bladder cancer (BCa). The aim of this study was to investigate the feasibility of segmenting the BCa region from bladder wall region on MRI, and quantitatively measuring the invasion depth of the tumor mass in bladder lumen for further clinical decision-making. This retrospective study involved 20 eligible patients with postoperatively pathologically confirmed BCa. It was conducted in the following steps: (1) a total of 1159 features were extracted from each voxel of both the certain cancerous and wall tissues with the T2-weighted (T2W) MRI data; (2) the support vector machine (SVM)-based recursive feature elimination (RFE) method was implemented to first select an optimal feature subset, and then develop the classification model for the precise separation of the cancerous regions; (3) after excluding the cancerous region from the bladder wall, the three-dimensional bladder wall thickness (BWT) was calculated using Laplacian method, and the invasion depth of BCa was eventually defined by the subtraction of the mean BWT excluding the cancerous region and the minimum BWT of the cancerous region.

**Results:**

The segmented results showed a promising accuracy, with the mean Dice similarity coefficient of 0.921. The “soft boundary” defined by the voxels with the probabilities between 0.1 and 0.9 could demonstrate the overlapped region of cancerous and wall tissues. The invasion depth calculated from proposed segmentation method was compared with that from manual segmentation, with a mean difference of 0.277 mm.

**Conclusion:**

The proposed strategy could accurately segment the BCa region, and, as the first attempt, realize the quantitative measurement of BCa invasion depth.

## Background

Bladder cancer (BCa) is the sixth-most common cancer in male worldwide [1–4]. It is estimated that 549, 000 new cases and 200,000 deaths occurred every year, with three-quarters of them occurring in men [1, 5]. Based on National Comprehensive Cancer Network (NCCN) guideline, surgical resection is one of the most effective treatment for BCa, while the invasion depth is one of the most important factors for choosing the optimal operation strategy like transurethral resection (TUR) of bladder tumor, partial resection or radical removal of bladder [6]. Therefore, preoperatively evaluating the invasion depth of BCa is very critical for the treatment-decision of patients with BCa.

As a routine method in the clinic, TUR biopsy can provide the prediction of invasion depth [7–10]. However, this approach is usually limited by the selection of the biopsy sites. It is reported that about 30–50% of the patients with BCa were down-staged after radical cystectomy [11]. With the development of medical imaging, an image-based approach may assess the tumor more comprehensively and avoid the risks of multiple biopsies [12]. Recently, several imaging studies using magnetic resonance imaging (MRI) have confirmed its benefit in predicting the aggressiveness of BCa [13], and in differentiating non-muscle-invasive (Stage ≤ T1) and muscle-invasive (Stage ≥ T2) BCa [14, 15], which might reveal the potential in predicting the invasion depth (staging) of BCa via MRI [16–18].

**Fig. 1:**
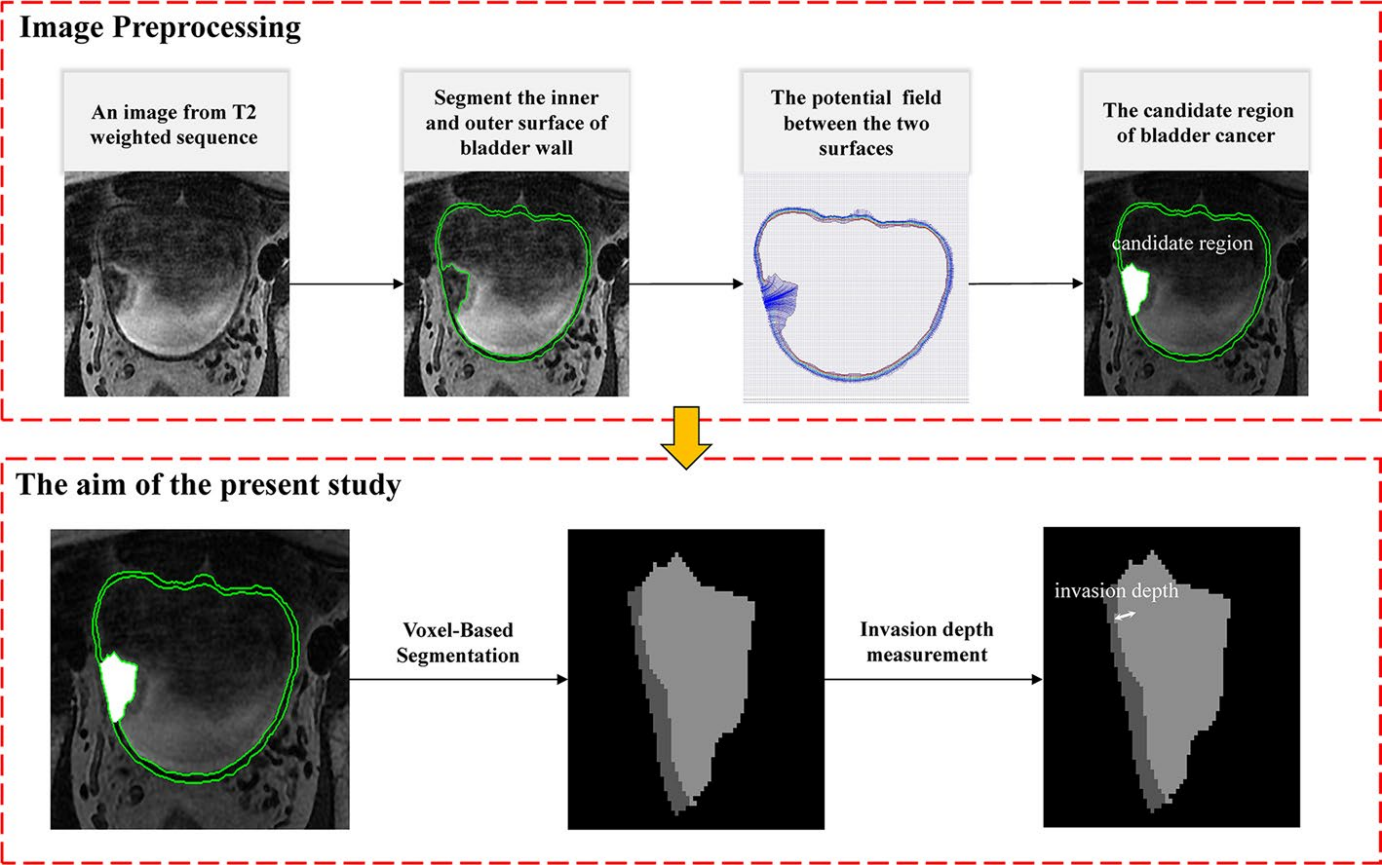
The proposed pipeline for the segmentation of the cancerous tissue and the measurement of the invasion depth of BCa

Specifically, in our previous studies, (1) a coupled directional level-set (CDLS) method was proposed to simultaneously segment the inner and outer surface of bladder wall on T2-weighted (T2W) magnetic resonance imaging (MRI) data [19], (2) three-dimensional (3D) thickness of bladder wall was calculated [20], to obtained the candidate region of BCa based on the variation of thickness and shape on T2W MRI [21]. However, it is difficult to further segment the certain cancerous region from the adherent wall tissue in the candidate region on T2W MRI (Fig. 1), due to the naturally weak boundary and similar intensity signals between the cancerous and wall tissues in this region.

In our recent study, a progressive dilated convolutional network was proposed to realize the simultaneous segmentation of multiple bladder regions, including the inner lumen, the wall region, the tumor masses and the background region outside the bladder [22]. However, one of the apparent limitations of this study is that the segmentation accuracy for tumor masses is less than satisfactory, with the Dice’s coefficient (DSC) of only 0.69 [22].

Due to the difficulty in precisely segmenting the BCa lesions from the adherent wall tissues, as far as we know, no study has currently taken a further look at how to quantitatively define and measure the invasion depth of bladder tumor on T2W images.

Therefore, the aim of this study was to first accurately segment the cancer region from the candidate region using the voxel-based features extracted from each voxel of cancerous and wall tissues, and then measure the invasion depth by using the Laplacian method to reflect the 3D surface alteration induced by BCa, as shown in Fig. 1. In the processing steps, the CDLS method was firstly used to segment the inner and outer surface of bladder wall, for the T2W images of each patient [19]. Between the inner and outer surfaces, the potential field and a streamline was generated, based on the Laplacian method [23], in which the BWT is the arc length of a streamline connecting a point on the inner surface and its corresponding point on the outer surface [20]. Then, the bent rate differences between the paired points were calculated to evaluate the bladder abnormalities caused by the lesions [21]. Based on these abnormal points, the candidate region of BCa can be obtained through the collection of all the voxels on the streamline, which is a mixed region containing both cancerous and wall tissues (Fig. 1). Based on the candidate region, the cancerous tissue can be segmented by a voxel-feature-based classification method proposed in this study. After subtracting the cancerous tissue from the entire bladder wall region, the invasion depth of BCa can be finally defined by the subtraction of the mean BWT excluding the cancerous region and the minimum BWT of the cancerous region.

## Results

### Demographics of the subjects

This study used an archived database of Tangdu hospital with 20 BCa patients postoperatively identified from October 2013 to August 2014. From each patient, the archived tumor lesion with the maximal size in bladder lumen was determined on the corresponding MRI dataset. These patients were randomly divided into a training set (*n* = 10) and a validation set (*n* = 10). Detailed information on the baseline demographics of these patients used in this study is shown in Table 1.

**Table 1:** Baseline demographics of the patients used in this study

Characteristics	Training	Validation
Patients, no. (%)	10 (50%)	10 (50%)
Age, median (range), years	66.5 (32, 79)	68.5 (53, 81)
Gender, no. (%)		
Male	8 (40%)	9 (45%)
Female	2 (10%)	1 (5%)
Tumor size, median (range), mm	21.69 (12.68, 39.94)	18.76 (10.16, 27.59)
Clinico-pathological stage, no. (%)		
Stage ≤ T1	4 (20%)	7 (35%)
Stage ≥ T2	6 (30%)	3 (15%)

### Optimal feature subset determined from the entire feature set

**Fig. 2:**
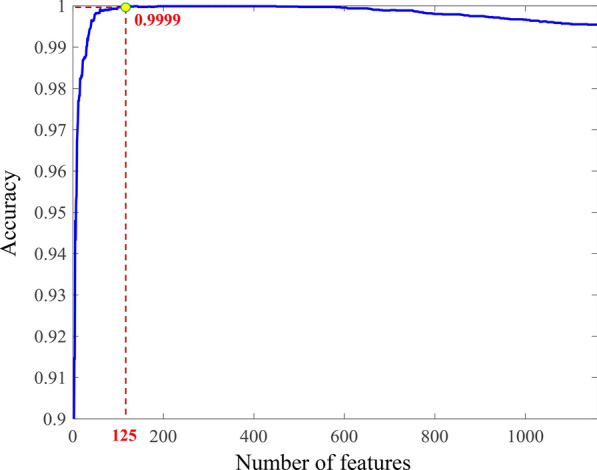
Optimal feature subset selection process

**Fig. 3:**
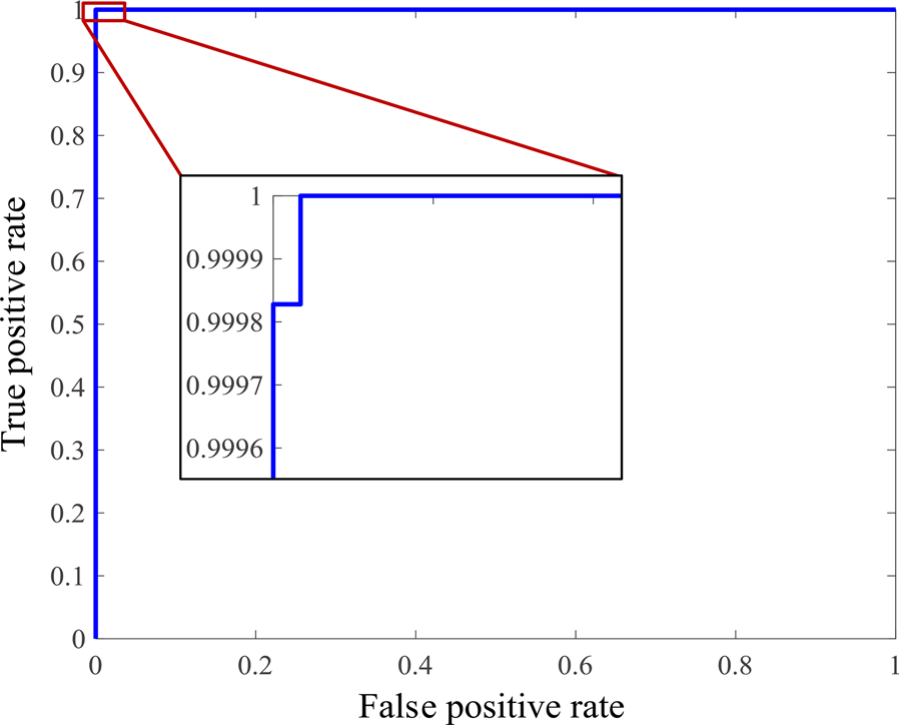
The ROC curve of the SVM classifier using the optimal feature subset

In this study, the training set contained 5812 cancerous voxels and 5851 wall voxels. A total of 1159 features were extracted from each voxel to characterize its properties. Considering that feature redundancy might actually exist and impair the capability of the classification model, feature selection was performed using support vector machine (SVM)-recursive feature elimination (RFE) approach [24–26]. The results are shown in Fig. 2. From the figure, we can see that when the number of features reached 125, the classification accuracy of the training set reached its highest value, with sensitivity = 99.98%, specificity = 1, accuracy = 99.99%, and AUC = 1 (Fig. 3). The subset composed of these top-ranked 125 features was treated as the optimal feature subset.

### Performance of the of cancerous region segmentation

**Fig. 4:**
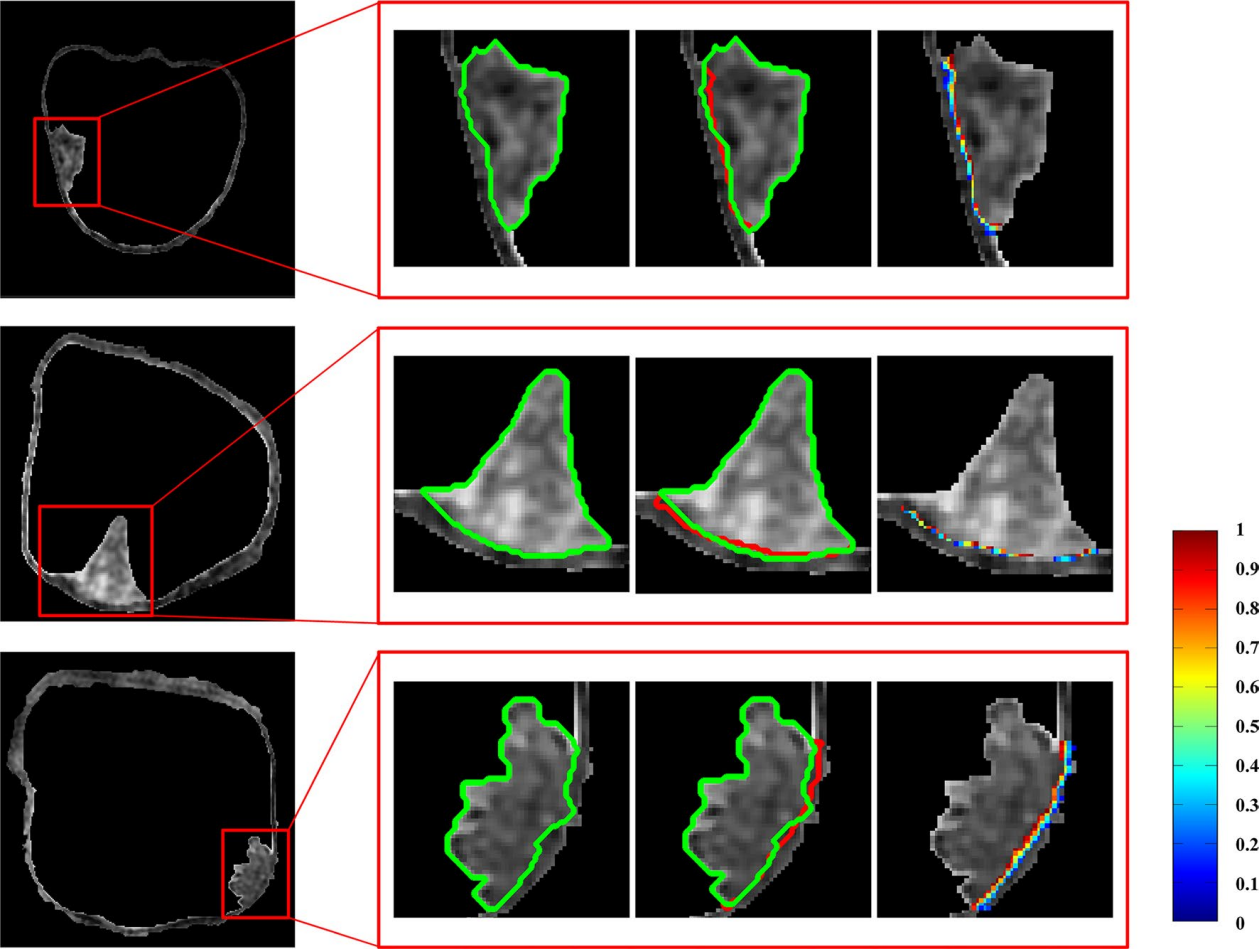
The segmentation results of bladder cancer. From left to right: the segmented results using CDLS method, ground truth (green color), segmented results of the proposed method (red color), and the “soft boundary”, a mixture region of cancerous and wall tissues, obtained by the proposed method

Using the optimal feature subset, 10 candidate regions obtained from the validation set were used to evaluate the accuracy of proposed method. Figure 4 shows the segmentation results for the first three subjects. From left to right: the segmented results using CDLS method, ground truth contoured using green color, segmented results of our method contoured using red color, and the results of “soft boundary” using our method. From Fig. 4, we can see that our results were well consistent with the manual delineation results. Meanwhile, defined by the probability value between 0.1 and 0.9, the “soft boundary” was identified, which is located at the interface between cancerous and wall tissues. This “soft boundary” indicated the overlapped or mixed regions of two types of tissues. Table 2 shows the DSC values for the testing subjects, which ranges from 0.848 to 0.985 with the average of 0.921, indicating its high consistence with manual delineation.

**Table 2:** The DSC value and the invasion depth calculated by using the validation set

Sample ID	DSC	*T*_ID_ (manual, mm)	*T*_ID_ (mm)	Difference (mm)
1	0.927	3.615	3.608	0.007
2	0.899	4.124	4.780	0.656
3	0.888	4.522	5.724	1.202
4	0.895	2.952	2.337	0.615
5	0.866	3.693	3.661	0.032
6	0.983	5.335	5.298	0.037
7	0.848	6.203	6.181	0.022
8	0.939	5.242	5.357	0.115
9	0.985	4.734	4.701	0.033
10	0.979	4.795	4.849	0.054
Mean ± SD	0.921 ± 0.050	–	–	0.277 ± 0.409

### Results of the invasion depth measurement

According to Table 2, columns 3 and 4 give the invasion depth of the validation set based on the manual segmentation and that using the proposed method, respectively. The differences between them are mostly lower than 1 mm, with the mean of 0.277 mm.

## Discussion

Invasion depth is an important index for treatment-decision of BCa, especially for the use of bladder-preserving or bladder-removing surgery [6]. To measure the invasion depth of BCa, a segmentation method using voxel-based features was firstly proposed to differentiate cancerous tissues from wall tissues and then a 3D thickness method was used to calculate the invasion depth quantitatively. To our knowledge, this is the first attempt to quantitatively measure the invasion depth. The preliminary results suggested that the proposed segmentation method could segment the BCa region accurately and the proposed pipeline could provide a quantitative measurement of invasion depth for treatment-decision of BCa.

The exact and robust segmentation of the BCa region is critical for the measurement of invasion depth. Due to the weak boundary between cancer region and wall region, it is hard to segment the cancer region only using the intensity value of MRI data. Considering the amplification characteristics of the imaging features [13], 1159 features were extracted from each voxel and an optimal feature subset containing top-ranked 125 features was obtained for the classification of cancerous and wall tissue. Using the optimal subset, we calculated the “hard” and “soft” boundary. The “hard” boundary is almost the same with the ground truth contoured by the radiologists, indicating that the extracted features can effectively distinguish cancer and wall tissues. Meanwhile, the “soft” boundary gathers at the interface between the cancerous and wall tissues, which could reflect the overlapped region of two tissues in images and should be considered in treatment.

Considering the 3D structure of bladder, we used the 3D thickness method to measure the invasion depth. Currently, no exact invasion depth of BCa can be obtained, thus, we compared our results with the invasion depth calculated from manual segmentation. Considering the voxel size was resampled to 1 × 1 × 1 mm^3^ when calculating the invasion depth, the error derived from a voxel may be 1 mm. Based on our initial testing, the difference of invasion depth derived from the manual segmentation and our proposed segmentation method is mostly lower than 1 mm, which further confirmed the accuracy of our segmentation method. Meanwhile, the proposed measurement method for invasion depth may provide a quantitative tool for treatment-decision of BCa.

Several limitations of this study should be addressed. Firstly, this is a retrospective study aiming to accurately segment bladder cancer on MRI and quantitatively estimate the invasion depth of the BCa, which needs multi-clinical validations and multi-modality data in future before the practical clinical applications. Secondly, the sample size of this study is small, which may influence the performance of classification and the evaluation of the segmentation results. Thirdly, only the T2W MRI sequence is included in this study. Currently, a semi-supervised classified method is under investigation using more datasets with multi-modality MRI, which takes the “soft boundary” into account and may further improve the segmentation.

## Conclusions

The proposed BCa segmentation method using the voxel-based feature can accurately segment the entire cancer region from the candidate region. As the first attempt, the quantitative measurement method of invasion depth may provide the quantitative information for the clinical decision.

## Methods

This retrospective analysis was ratified by the institutional Ethics Review Board, and the requirement for informed content was waived.

### Subject enrollment

The database contains 20 BCa patients identified from October 2013 to August 2014. All patients were scanned by a 3.0-T MRI scanner (Discovery MR 750; GE Medical Systems) from the Tangdu Hospital. The inclusion criteria were as follows: (1) patients with pathologically confirmed BCa lesions after operation, (2) the maximal lesion in bladder lumen and its postoperatively pathological findings were archived, and (3) T2W MRI sequence was performed prior to any treatment. The MRI data with poor imaging quality were excluded, which may make the accurate bladder carcinoma segmentation difficult. The T2W sequence (GE Discovery MRI 750 3.0 T) was performed to obtain the preoperative bladder images of each patient, and the main parameters of this sequence included repetition time of 2500 ms, echo time of 135 ms, slice thickness of 1 mm, and pixel size of 0.5 × 0.5 mm^2^. These patients were then randomly divided into the training set for model development and the validation set for performance assessment, with 10 patients in each set.

### Candidate region determination

For T2W MRI sequence of each patient, the CDLS method was used to segment the inner and outer surface of bladder wall [19]. Between the two surfaces, a potential field and a streamline can be generated based on the Laplacian method [23], in which the thickness of bladder wall is the arc length of a streamline connecting a point on the inner surface and its corresponding point on the outer surface [20]. The bent rate differences between the paired points that reflect bladder abnormalities are caused by lesions [21]. From these abnormal points, all the voxels on the streamline can constitute a candidate region of BCa, as shown in Fig. 1, which usually contains both cancerous and wall tissues to be separated.

### Voxel-feature-based segmentation of BCa region

After the previous processing (Fig. 1), the bladder wall and the candidate region of each patient can be obtained. In the training set, the cancerous and wall tissues were manually delineated, and 1159 features were extracted from each voxel of them. Then the SVM-RFE method was adopted to first select an optimal subset of features and then distinguish the cancerous and the wall tissues from the voxels [27]. Using the model constructed by the optimal feature subset in the training set, 10 candidate regions obtained from the validation set were used to evaluate the accuracy of proposed method.

#### (1) Volume of interest delineation from training set

**Fig. 5:**
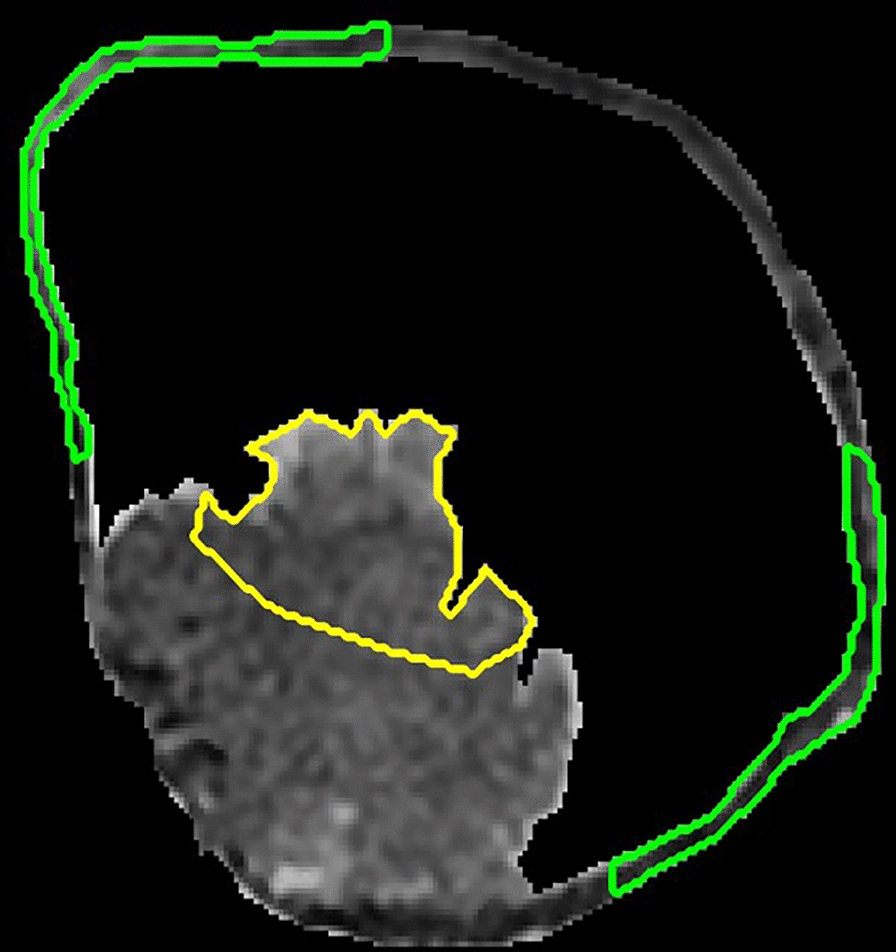
Cancerous and wall VOIs delineation of the training dataset. The cancerous VOI is outlined by the yellow contour. The wall VOI is contoured in the green

In the training set, volumes of interest were previously manually contoured by a radiologist who has 8 years of bladder MRI reading experience. The cancerous VOIs were contoured within the candidate region and away from the bladder wall as much as possible, as described as the yellow contour in Fig. 5. Due to limited voxels and weak boundary between the cancerous and wall tissues, we selected wall voxels near the candidate region as the wall VOIs and tried to keep its number of voxels approximately equal to that of cancerous VOIs.

#### (2) Voxel-based feature extraction

Previous studies indicate that intensity and texture features could reflect pathological properties of different tissue types [28, 29], which can be used to distinguish the BCa tissues from wall tissues [30]. Prior to intensity and texture features extraction, the wavelet transform was used to decompose the original image to obtain 16 wavelet images. Thus, a total of 17 images (16 wavelet images and the original image) were used to extract the intensity and texture features.

The intensity features describe the intensity information of the target voxel (*x*, *y*, *z*) and its six neighbor voxels (*x* − 1, *y*, *z*), (*x* + 1, *y*, *z*), (*x*, *y* − 1, *z*), (*x*, *y* + 1, *z*), (*x*, *y*, *z* − 1), (*x*, *y*, *z* + 1). A total of 20 intensity description features were extracted, which contains the intensities of these seven voxels, the mean intensity-values of 3 × 3 areas centered at the seven voxels, respectively, and the intensity-differences between the target voxel and its six neighbor voxels, respectively.

In this study, the Leung–Malik (LM) filter bank was used to extract texture features [31]. The LM filter bank consists of 48 filters, which includes 18 first derivatives and 18 second derivatives of Gaussian-differential filters (6 orientations, 3 scales), 8 Laplacian of Gaussian filters, and 4 Gaussian smoothing filters. The response from 48 filters is taken as 48 texture features for each voxel.

By considering the *x*, *y*, and *z* coordinate values of each voxel as 3 location features, in this study, a total of 1159 features were generated for each voxel, i.e., 3 location features + (20 intensity features + 48 texture features) × 17 images.

#### (3) Feature selection and classification using the SVM

Among features obtained from each voxel, some may be correlated and redundant, which may affect the classification performance [30, 32, 33]. In the present study, we used the SVM-RFE method implemented by LIBSVM package [34], to find the optimal feature subset with the best differentiation performance [35]. After each iteration, the feature with smallest absolute weight was eliminated. Finally, the optimal feature subset was determined using this approach and a fivefold cross-validation, which contains the first ***N*** features with the highest mean accuracy. The classification performance was evaluated by the sensitivity, specificity, accuracy, and area under the curve (AUC) of the receiver operating characteristics (ROC).

#### (4) The segmentation of the cancerous tissues from the candidate regions

Using the optimal feature subset, the SVM prediction model was performed on the validation set. Based on the SVM model, we can obtain the probability value of each voxel belonging to the cancer region. According to the probability, we calculated the “hard” and “soft” boundary to distinguish the cancer and wall regions.

To obtain the “hard boundary”, we used the probability of 0.5 as the threshold, and then segmented the cancer region from the wall region within the candidate region. After classification, a postprocessing, including the maximum connected region (max-region) and void filling, was performed to obtain the continuous boundary. Meanwhile, according to the position of concerned voxel, the “soft boundary” was defined by the probabilities between 0.1 and 0.9, calculated by the SVM prediction model.

#### (5) The accuracy evaluation of proposed segmentation method

In this study, the manual segmentation was treated as the ground truth. The contours of the cancer regions from the validation set were drawn by another two radiologists with 9 years of experience in MRI interpretation. After delineation of each cancer region slice by slice independently, they worked together on the contours according to a consensus reading. The DSC was used to quantitatively evaluate the performance of proposed segmentation method, which can be calculated by *DSC*(*S*_*G*_, *S*_*A*_) = 2 ×|*S*_*G*_ ∩ *S*_*A*_|/(|*S*_*G*_| +|*S*_*A*_|), where *S*_*G*_ denotes the manual segmentation of radiologists and *S*_*A*_ denotes segmentation results from our method.

### Measurement of invasion depth

**Fig. 6:**
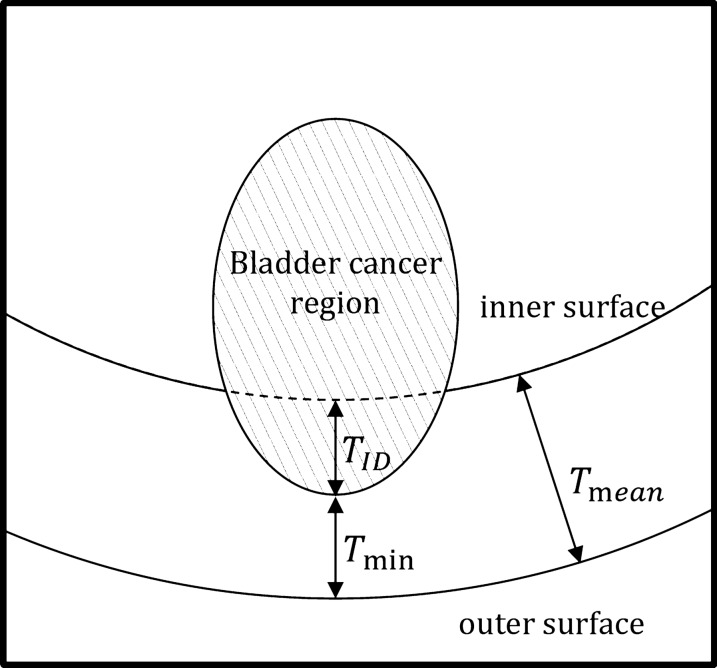
The diagram for the measurement of invasion depth. *T*_mean_: the mean thickness of bladder wall excluding the cancer region, *T*_min_: the minimum thickness of the cancer region, *T*_ID_: invasion depth calculated by *T*_mean_ − *T*_min_

Based on the segmented results, the cancer region was excluded from the candidate region. After that, the 3D thickness map of the bladder wall was calculated using the Laplacian method [20]. In the 3D thickness map, the average thickness of bladder wall *T*_mean_ was defined by the mean thickness of bladder wall excluding the candidate region to avoid any bias induced by the cancer region, and the minimum thickness of the candidate region *T*_min_ was obtained. In this way, the invasion depth (*T*_ID_) can be evaluated by *T*_mean_ − *T*_min_, as shown in Fig. 6.

Due to the limitation of tissue biopsies, the exact invasion depth of a BCa could not be obtained. Instead, the value of invasion depth calculated from the proposed segmentation results was compared with that from manual segmentation.

### Data statement

The datasets in this study are currently not available for free public access owing to patient privacy concerns, but may be obtainable from the corresponding authors on reasonable request approved by the institutional review boards.

## Data Availability

The data sets used and/or analyzed during the current study are available from the corresponding author on reasonable request.

## Abbreviations

AI: Artificial intelligence
AUC: Area under the curve
BCa: Bladder cancer
BWT: Bladder wall thickness
CDLS: Coupled directional level-set
DSC: Dice’s coefficient
LM: Leung–Malik
MRI: Magnetic resonance imaging
NCCN: National Comprehensive Cancer Network
ROC: Receiver operating characteristics
SVM-RFE: Support vector machine-based recursive feature elimination
3D: Three dimensional
T2W: T2-weighted
TUR: Transurethral resection
VOI: Volume of interested.
